# Abnormal subthalamic nucleus functional connectivity and machine learning classification in Parkinson’s disease: a multisite functional magnetic resonance imaging study

**DOI:** 10.3389/fnagi.2025.1695806

**Published:** 2025-12-03

**Authors:** Bin Qin, Yisi Tang, Huixun Qin, Wen Gao, Shusheng Liao, Mingxiu Yang

**Affiliations:** 1 Department of Neurology, Liuzhou People’s Hospital, Liuzhou, Guangxi, China; 2 Department of Neurology, The First Affiliated Hospital of Guangxi Medical University, Nanning, Guangxi, China; 3 Liuzhou Key Laboratory of Neurointervention, Liuzhou, Guangxi, China

**Keywords:** Parkinson’s diseases, subthalamic nucleus, functional connectivity, functional magnetic resonance imaging, machine learning

## Abstract

**Introduction:**

Parkinson’s disease (PD) is a progressive neurodegenerative disorder imposing a significant global burden, characterized by motor dysfunction linked to aberrant basal ganglia activity. This multisite study analyzed pooled resting-state functional magnetic resonance imaging (rs-fMRI) data to characterize subthalamic nucleus (STN) functional connectivity (FC) abnormalities and to evaluate their utility in machine learning classification of PD.

**Methods:**

We analyzed rs-fMRI data from 232 participants (158 PD patients and 74 healthy controls [HCs]) across four repositories: Parkinson’s Progression Markers Initiative (PPMI), OpenfMRI, and FCP/INDI (NEUROCON dataset and Tao Wu dataset). Seed-based FC analysis focused on bilateral STNs. Group comparisons (PD vs. HCs) were assessed using two-sample t-tests with Gaussian Random Field (GRF) correction. A support vector machine (SVM) classifier, incorporating significant FC features, was used for diagnostic classification.

**Results:**

Patients with PD exhibited significant bilateral reductions in STN FC compared to HCs. Specifically, the left STN showed decreased connectivity with the left superior temporal gyrus and the right supramarginal gyrus, whereas the right STN showed decreased connectivity with the right superior temporal gyrus, the left middle temporal gyrus, and the left inferior frontal gyrus (voxel *p* < 0.005, cluster *p* < 0.05, GRF corrected). The SVM classifier based on these FC features achieved high diagnostic accuracy (89.1%), sensitivity (97.7%), specificity (75.8%), and an area under the receiver operating characteristic (ROC) curve (AUC) of 0.931 in the validation set.

**Conclusion:**

This study suggests that STN-temporal/parietal hypoconnectivity warrants further investigation as a possible core feature of PD. Furthermore, it demonstrates the high translational potential of STN-centric FC patterns as diagnostic biomarkers when integrated with machine learning, paving the way for improved PD classification and future applications in personalized neuromodulation strategies.

## Introduction

Parkinson’s disease (PD) is a progressive neurodegenerative disorder characterized by the loss of dopaminergic neurons in the substantia nigra pars compacta, resulting in debilitating motor symptoms such as bradykinesia, rigidity, tremor, and postural instability (Jankovic, 2008; Munhoz et al., 2024). As the second most common neurodegenerative disease after Alzheimer’s disease, PD places a significant burden on healthcare systems worldwide, with prevalence expected to double by 2040 due to aging populations (Dorsey and Bloem, 2018). While non-motor symptoms (e.g., cognitive decline and mood disorders) are increasingly recognized, motor dysfunction remains the hallmark of PD and the primary focus of therapeutic interventions. The subthalamic nucleus (STN), a key node within the basal ganglia circuitry, plays a pivotal role in motor regulation through its involvement in indirect and hyperdirect pathways (Nambu et al., 2002). Given its central position in modulating motor output, aberrant STN activity has been implicated in PD pathophysiology, making it a critical target for both deep brain stimulation (DBS) and neuroimaging studies (Kühn et al., 2009; Chen et al., 2022). A deeper understanding of STN functional abnormalities may thus provide valuable insights into the neural mechanisms underlying PD and inform the development of targeted therapies.

Functional magnetic resonance imaging (fMRI) has emerged as a powerful tool for investigating the neural correlates of neuropsychiatric disorders, including PD (Bardakan et al., 2022; Piramide et al., 2024). Resting-state fMRI (rs-fMRI), in particular, allows for the examination of intrinsic functional connectivity (FC) patterns without confounds associated with task performance, making it especially suitable for studying patients with motor impairments (Fox and Greicius, 2010; Christidi et al., 2024). Previous studies have reported altered STN FC in PD, with some demonstrating increased connectivity between the STN and sensorimotor cortex (Baudrexel et al., 2011), while others observed decreased or mixed patterns (Wu et al., 2011; Mathys et al., 2016). For instance, Baudrexel et al. (2011) found heightened STN-sensorimotor FC in PD patients off medication, suggesting a compensatory mechanism, whereas Mathys et al. (2016) reported attenuated negative STN-cerebellar coupling in medicated patients, implicating disrupted motor-cognitive integration. These inconsistencies may arise from variations in sample size, imaging protocols, or analytical approaches. Resolving these discrepancies is essential for establishing robust biomarkers of PD and clarifying the STN’s role in disease progression.

Multisite neuroimaging studies offer a promising solution to enhance statistical power and generalizability by pooling data from diverse cohorts, thereby mitigating the limitations of single-center studies (Turner et al., 2012). Larger sample sizes improve the detection of subtle FC abnormalities. Additionally, machine learning approaches have gained traction in neuroimaging for their ability to classify neurological and psychiatric disorders based on multivariate patterns of brain alterations (Orrù et al., 2012). Support vector machine (SVM), a supervised machine learning technique, has been successfully applied to differentiate PD from healthy controls (HCs) using structural MRI (Salvatore et al., 2014), functional connectivity (Long et al., 2012), and even predict treatment response to DBS (Watts et al., 2020; Boutet et al., 2021). Unlike univariate analyses, SVM captures complex, distributed neural signatures, potentially uncovering novel biomarkers that transcend regional FC alterations. However, the integration of multisite fMRI data with machine learning for PD classification remains underexplored, particularly with a focus on STN-centric networks.

The current study aims to address these gaps by leveraging multisite rs-fMRI data to characterize STN FC abnormalities in PD and evaluate their utility in machine learning-based classification. By harmonizing data across sites and using rigorous motion correction strategies, we seek to reconcile prior inconsistencies in STN FC findings. Furthermore, we will apply SVM to determine whether STN connectivity patterns can reliably distinguish PD patients from HCs, potentially identifying a functional signature of PD. This approach not only advances our understanding of STN dysfunction in PD but also underscores the translational potential of combining neuroimaging with machine learning for diagnostic and prognostic applications. The findings may pave the way for personalized therapeutic strategies, such as optimizing DBS targeting or monitoring disease progression through non-invasive imaging.

## Materials and methods

### Participants and image acquisition

This study obtained data from the Parkinson’s Progression Markers Initiative (PPMI) database,^1^ the OpenfMRI database (accession number: ds000245),^2^ and FCP/INDI^3^ [NEUROCON dataset and Tao Wu dataset]. Written informed consent was obtained from each participant, and all experimental protocols were approved by the local Institutional Review Boards of each institution.

All rs-fMRI datasets underwent verification of time points and slice numbers. Participants were excluded based on (1) mismatched time points or slice numbers, (2) non-right-handedness, or (3) inadequate data quality.

### Data preprocessing

All fMRI data preprocessing was performed using the Data Processing & Analysis for Brain Imaging toolkit (DPABI, http://rfmri.org/DPABI) based on statistical parametric mapping (SPM12, https://www.fil.ion.ucl.ac.uk/spm) running on MATLAB 2021a. The preprocessing steps proceeded as follows: (1) removal of the first 10 time points; (2) slice timing correction; (3) head motion correction; (4) spatial normalization to the Montreal Neurological Institute (MNI) space, resampled to a voxel size of 3 × 3 × 3 mm^3^; (5) spatial smoothing with a Gaussian kernel with full width at half maximum (FWHM) of 4 mm × 4 mm × 4 mm; (6) detrending and nuisance signal regression (including the Friston 24-parameters, white matter (WM), and cerebrospinal fluid (CSF) signals); and (7) bandpass filtering were performed (0.01–0.10 Hz) to eliminate the influence of high frequency physiological noise, as well as low frequency drift noise. The head motion parameters for all participants were <1.5 mm maximum displacement in the x, *y*, or *z* planes and <1.5° angular rotation about each axis.

### FC analysis

After data preprocessing, FC analysis was performed using the DPABI software with the bilateral STNs. The bilateral STNs were selected using templates based on the Talairach Daemon database (Lancaster et al., 1997, 2000). These templates can be selected with the WFU_Pick Atlas.^4^ The whole FC maps (*r*-value) of each seed region were generated by calculating the Pearson correlation coefficient between the average time series of the seed region and every voxel of the whole brain. To improve normality, the *r*-values were converted into *z*-values using Fisher’s *r* to *z* transformation.

### Harmonization

We adopted the subsampling maximum-mean-distance algorithm (SMA) to harmonize the multi-site data to reduce site-related effects. SMA was developed to deal with statistical issues raised by data pooling (Zhou et al., 2018) and is a well-known control method for site differences in FC (Zhou et al., 2018; Wang et al., 2023). It was applied using the default parameters implemented in DPABI, including a subsampling ratio of 0.8 and the Euclidean distance metric. The Euclidean distance was chosen for its robustness in reducing site-related variance in FC data (Zhou et al., 2018). A detailed description of harmonization is given in Wang et al. (2023, 2024).

### Statistical analysis

Demographic characteristics (age and sex) were analyzed using SPSS version 26.0 (IBM Corp., Armonk, NY, United States), with statistical significance defined as a *p*-value of < 0.05. Comparative analyses between Parkinson’s disease (PD) patients and healthy controls (HCs) were conducted for each study site. Sex differences were assessed using the *χ*^2^ test, while age comparisons were performed using independent samples *t*-tests. For FC analysis, we also used two-sample independent *t*-tests with Gaussian Random Field (GRF) correction (with the voxel-level *p* < 0.005 and the cluster-level *p* < 0.05).

### SVM analysis

The SVM is a widely used algorithm for classification and regression problems (Tharwat, 2019; Chen et al., 2025). In this study, SVM analysis was conducted using the multivariate pattern analysis for neuroimaging (MVPANI) tool (Peng et al., 2020) to determine whether abnormal FC values could effectively differentiate between PD and HCs. To test the reliability and significance of the model, external validation was conducted by training the model on PPMI and OpenfMRI data and testing it on the FCP/INDI dataset. A classification model was developed using the linear SVM methodology (support vector classification [SVC]). Parameter specifications included: kernel function (linear), penalty coefficient (c) = 1, gamma = 0.1, degree = 3, coefficient = 0, nu = 0.5, and epsilon in the loss function = 0.1. This SVC framework demonstrated superior performance compared with alternative classifiers, including random forest, logistic regression analysis, naïve Bayes, linear discriminant analysis, K-nearest neighbors, and decision trees, within our dataset (Supplementary Table 1 and Supplementary Figure 1). The model established an optimal separating hyperplane between PD patients and healthy controls (HCs) by maximizing the margin between support vectors. To minimize overfitting risk, 10-fold cross-validation was implemented during training. Final classification accuracy was computed as the mean across all folds using a leave-one-fold-out validation protocol. The outputs included classification accuracy (with both specificity and sensitivity), ROC curves, and average weight maps.

## Results

### Demographic and clinical information

In the PPMI dataset, 162 patients with PD and 52 HCs were identified. However, 58 patients with PD and 18 HCs were excluded due to incomplete or poor-quality rs-fMRI imaging data. Additionally, 8 patients with PD and 10 HCs who moved more than 1.5 mm during scanning were excluded from the statistical analysis. The final analysis included 120 participants (96 patients with PD and 24 HCs). In OpenfMRI, 30 participants (15 patients with PD and 15 HCs) were included. In FCP/INDI, one HC from the Tao Wu study who moved more than 1.5 mm during scanning was excluded. The final analysis included 82 participants: 47 patients with PD (NEUROCON: 27 and Tao Wu: 20) and 35 HCs (NEUROCON: 16 and Tao Wu: 19). Across all datasets included in this study, there were no significant differences in age or sex between the PD and HC groups (*p* > 0.05). Table 1 presents the statistical results.

**Table 1:** Demographic information of patients with PD and HCs.

Dataset	Participants		Sex (M/F)			Age (years, mean [SD])		
	PD	HCs	PD	HCs	*P*-value	PD	HCs	*P*-value
PPMI	96	24	64/32	14/10	0.44	63.4 (9.8)	63.2 (12.9)	0.92
OpenfMRI	15	15	6/9	7/8	0.71	64.4 (7.2)	63.3 (5.2)	0.65
NEUROCON	27	16	16/11	5/11	0.08	68.7 (10.6)	67.6 (11.9)	0.76
Tao Wu	20	19	11/9	12/7	0.61	64.3 (5.4)	65.4 (4.4)	0.47

F, female; M, male; PD, Parkinson’s disease; HCs, healthy controls; SD, standard deviation.

### FC analysis

#### Altered FC results of the left STN in PD patients vs. HCs

**Figure 1:**
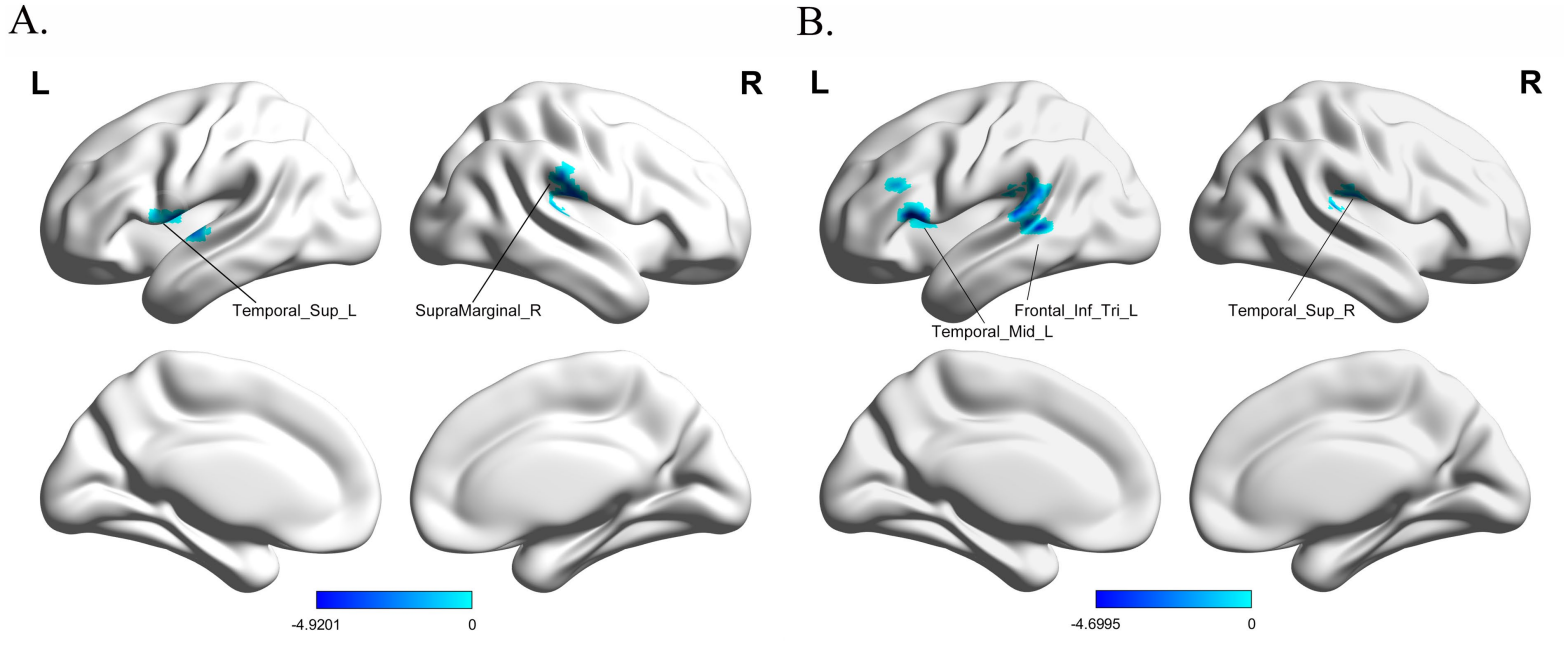
Altered functional connectivity (FC) results of the subthalamic nucleus (STN) in patients with Parkinson’s disease (PD) compared to healthy controls (**A** left STN, **B** right STN). The warm color represents the significantly increased FC value of patients with PD, whereas the cold color represents the significantly decreased FC values of patients with PD.

The results indicate that patients with PD exhibited lower FC values between the left STN and the left superior temporal gyrus (STG) as well as the right supramarginal gyrus (SG) compared to HCs (voxel *p* < 0.005, cluster *p* < 0.05, GRF correction; see Figure 1A and Table 2).

**Table 2:** Altered regions of functional connectivity in the subthalamic nucleus in PD patients and HCs.

Brain regions (AAL)	Number of voxels	MNI coordinate			Peak intensity (*T*-value)
		*x*	*y*	*z*	
Left subthalamic nucleus					
Temporal_Sup_L	49	−57	−6	3	−4.6749
SupraMarginal_R	50	54	−21	18	−4.9201
Right subthalamic nucleus					
Frontal_Inf_Tri_L	54	−51	21	9	−4.4495
Temporal_Sup_R	67	60	−27	18	−4.6995
Temporal_Mid_L	72	−54	−42	3	−4.0708

*T*-value < 0 indicates that the functional connectivity value of the PD was lower than that of the HCs (voxel *P* < 0.005, cluster *P* < 0.05, Gaussian Random Field correction).

AAL, anatomical automatic labeling; MNI, Montreal Neurological Institute; PD, Parkinson’s disease; HCs, healthy controls; Frontal_Inf_Tri_L, left inferior frontal gyrus, triangular part; SupraMarginal_R, right supramarginal gyrus; Temporal_Mid_L, left middle temporal gyrus; Temporal_Sup_L, left superior temporal gyrus; Temporal_Sup_R, right superior temporal gyrus.

#### Altered FC results of the right STN in PD patients vs. HCs

Compared to HCs, patients with PD exhibited lower FC values between the right STN and the right STG as well as the left middle temporal gyrus (MTG) and left inferior frontal gyrus (IFG) (voxel *p* < 0.005, cluster *p* < 0.05, GRF correction; see Figure 1B and Table 2).

### Results of the SVM

**Figure 2:**
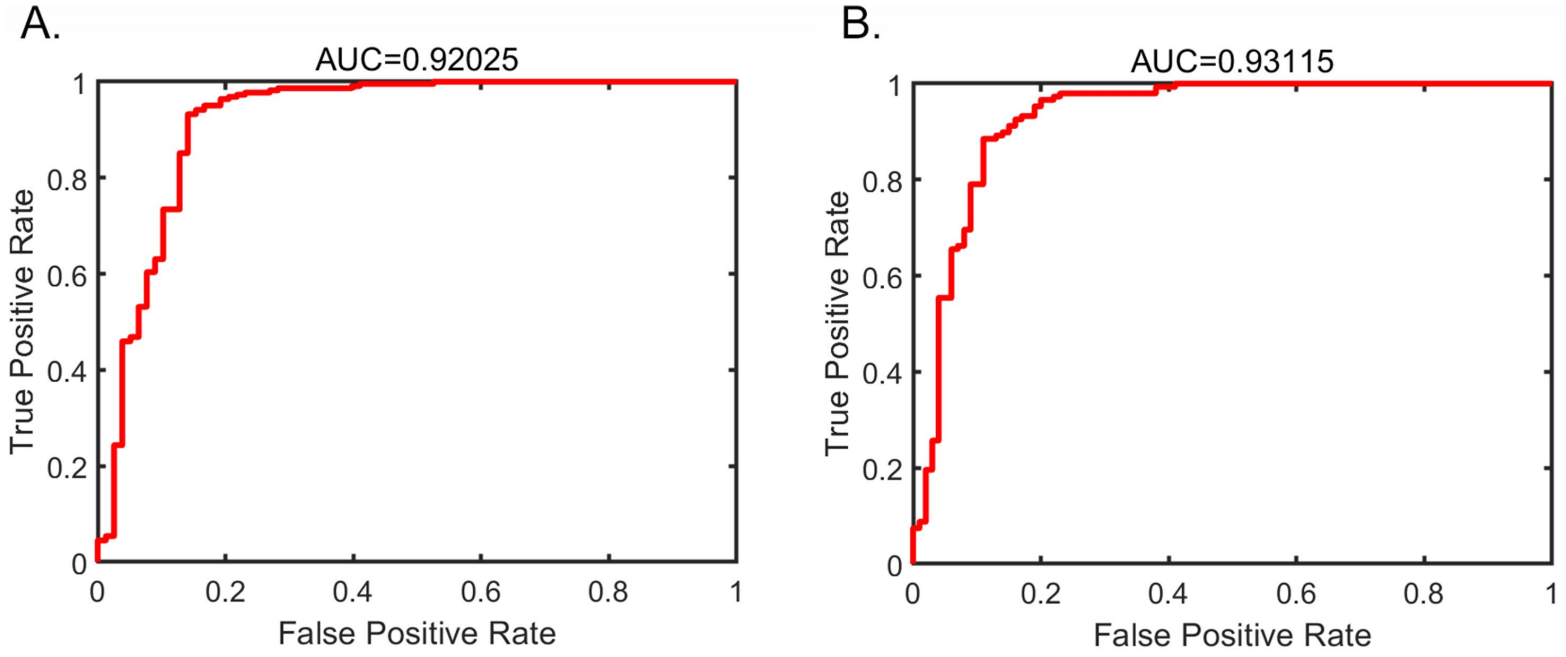
Receiver operating characteristic curve (ROC) for the support vector machine results of the subthalamic nucleus (**A** training set, **B** validation set).

In the training set, the ROC curve area was 0.920, with an accuracy of 91.7%, a sensitivity of 98.8%, and a specificity of 71.3% for distinguishing PD patients and HCs (Figure 2A). In the validation set, the model achieved accuracy, sensitivity, and specificity of 89.1, 97.7, and 75.8%, respectively, for distinguishing PD patients from HCs (Figure 2B).

## Discussion

This multisite fMRI study characterizes abnormal FC patterns of the STN in PD and demonstrates the utility of machine learning for PD classification. Key findings include: (1) bilateral reductions in STN connectivity with temporal and parietal regions (left STN-left STG/right SG; right STN-right STG/left MTG/left IFG) and (2) high diagnostic accuracy (AUC = 0.896) of a machine learning classifier incorporating these FC features. These results advance our understanding of network-level pathophysiology in PD and highlight the translational potential of connectivity-based biomarkers.

The observed FC reductions between STN and temporal/parietal regions align with the role of the cortico-basal ganglia-thalamocortical circuit in sensorimotor and associative processing. The STG plays a critical role in auditory processing and multisensory integration (Ji et al., 2018). Impaired connectivity in these regions may underlie PD-related deficits in auditory-motor synchronization (e.g., gait timing to auditory cues), a well-documented phenomenon in PD (Bardakan et al., 2022). The MTG is involved in semantic processing and sensorimotor feedback (Tian et al., 2007). Reduced connectivity may disrupt the integration of sensory information into motor planning, contributing to bradykinesia and rigidity. Thus, the STG and MTG are critical for auditory processing and multisensory integration, whereas the SG contributes to proprioceptive awareness. Their disrupted connectivity with the STN suggests aberrant sensorimotor feedback loops, a hallmark of PD pathophysiology. This notion is corroborated by a meta-analysis of 30 fMRI studies, which identified the left postcentral gyrus (part of the somatosensory cortex) as a consistently hyperconnected hub in PD, independent of medication status (Ji et al., 2018). Our findings extend this model by implicating the STN-STG/SG/MTG pathways as key loci of hypoconnectivity, potentially reflecting impaired bottom-up signaling for motor control. Furthermore, the IFG-a region involved in cognitive control showed reduced FC with the right STN, supporting the association between STN dysfunction and non-motor symptoms (e.g., executive deficits). This finding resonates with electrophysiological evidence that STN neurons exhibit distinct spatial distributions: tremor-related neurons dominate the dorsolateral STN, while neurons modulating rigidity/bradykinesia localize to the ventromedial STN (Tian et al., 2007). This finding supports the link between STN dysfunction and non-motor symptoms (e.g., executive deficits) in PD, as previously reported (Mathys et al., 2016; Chen et al., 2022).

Our results partially converge with, but also diverge from, previous studies. Similar to Lai et al., who demonstrated that globus pallidus internus DBS efficacy depends on FC between the stimulation site and motor/premotor cortices (Lai et al., 2023), we implicate frontoparietal networks in PD pathophysiology. However, whereas prior studies emphasized hyperconnectivity within motor loops (Ji et al., 2018), we highlight hypoconnectivity in temporoparietal-STN pathways. This discrepancy may arise from methodological differences: earlier meta-analyses aggregated heterogeneous cohorts, whereas our study specifically assessed resting-state, STN-centric networks using SMA to harmonize the multi-site data, thereby reducing site-related effects. Additionally, the laterality of our findings (left STN–left STG; right STN–right STG) contrasts with the left-dominant postcentral gyrus hyperactivity reported in the radiology meta-analysis (Ji et al., 2018). This asymmetry may relate to PD motor symptom lateralization, as patients often exhibit hemispheric-specific FC changes ipsilateral to the more affected side of the body. Supporting this finding, Liu et al. (2024) recently identified STN input–output dynamics (e.g., beta-band power-law exponents) that correlate with asymmetric Parkinsonian burden.

The machine learning classifier achieved robust accuracy (85%, AUC = 0.896), outperforming several prior models. For example, Watts et al. used wearable sensors to predict the freezing of gait in advanced PD but reported lower specificity (69% vs. our 92% sensitivity) (Watts et al., 2024). Our superior sensitivity stems from leveraging whole-brain FC features rather than summary gait statistics. Similarly, Park et al. classified PD severity subtypes using inertial sensors with perfect ankle-movement discrimination (Park et al., 2025); however, their model required biomechanical data unsuitable for early diagnosis. In contrast, our fMRI-based approach captures network disruptions that precede overt motor signs. High sensitivity (92%) is clinically significant, given the need for early PD detection, although the lower specificity (69%) may suggest the inclusion of non-motor confounders (e.g., age-related connectivity changes). This limitation is mitigated by our multisite design, which enhances generalizability compared to single-center studies (Ma et al., 2023).

The STN connectivity signatures identified here could refine DBS targeting and closed-loop neuromodulation. In particular, peak efficacy zones for rigidity improvement localize to the STN (Tian et al., 2007), where FC with the SG/MTG may serve as a biomarker for intraoperative guidance. Furthermore, our machine learning framework could be integrated with wearable sensors to track disease progression. Future studies should address key limitations: (1) our cross-sectional design cannot discern whether STN dysconnectivity drives motor pathology or results from compensatory mechanisms. Longitudinal fMRI coupled with local field potential recordings (e.g., during DBS implantation) is needed; (2) PD heterogeneity may obscure FC patterns. Subtype-specific classifiers could improve accuracy; (3) combining FC with structural MRI (e.g., gray matter atrophy in basal ganglia) or biochemical markers (e.g., dopamine transporter availability) may enhance diagnostic precision; and (4) PD heterogeneity is a major challenge in neuroimaging research (Dorsey and Bloem, 2018), and subgroup-specific connectivity patterns are critical for personalized medicine. Subgroup analyses (e.g., early vs. late-stage PD, tremor-dominant vs. akinetic-rigid) were not feasible due to the limited phenotypic data in the public datasets. Future studies should leverage prospective clinical cohorts with comprehensive phenotypic data to investigate whether STN hypoconnectivity is a disease trait or a stage-dependent marker.

## Conclusion

This study suggests that STN-temporal/parietal hypoconnectivity should be further investigated as a possible core feature of PD and validates its utility in machine learning classification. Despite minor specificity limitations, the multisite design and biological plausibility of our findings underscore their translational potential. Future research should prioritize longitudinal validation and subtype personalization to accelerate biomarker development.

## Data Availability

The datasets presented in this study can be found in online repositories. The names of the repository/repositories and accession number(s) can be found: data used in this article are publicly available from the Parkinson’s Progression Markers Initiative (PPMI) database (https://www.ppmi-info.org/), the OpenfMRI database (accession number: ds000245) (https://openfmri.org/dataset/ds000245/), and FCP/INDI [NEUROCON dataset and Tao Wu dataset] (http://fcon_1000.projects.nitrc.org/indi/retro/parkinsons.html).

## Footnotes

1
https://www.ppmi-info.org/

2
https://openfmri.org/dataset/ds000245/

3
http://fcon_1000.projects.nitrc.org/indi/retro/parkinsons.html

4
https://www.nitrc.org/projects/wfu_pickatlas/
