# Supplementary material for: Abnormal subthalamic nucleus functional connectivity and machine learning classification in Parkinson’s disease: a multisite functional magnetic resonance imaging study
Source: Front Aging Neurosci. 2025 Dec 3;17:1695806. doi: 10.3389/fnagi.2025.1695806 (PMC12708609; doi:10.3389/fnagi.2025.1695806)
Supplement: Supplementary file 1 [file Table_1.docx]

**Supplementary TABLE 1 The results of Multiple classification model (in training set)**

| **Model** | **Specificity (%)** | **Sensitivity (%)** | **Accuracy (%)** | **AUC** |
| --- | --- | --- | --- | --- |
| Random forest | 22.5 | 98.6 | 79.0 | 0.706 |
| Logistic regression | 66.9 | 99.0 | 90.0 | 0.908 |
| Naïve Bayes | 40.2 | 74.3 | 65.7 | 0.589 |
| Linear discriminant analysis | 64.9 | 97.3 | 88.7 | 0.897 |
| K-nearest neighbors | 17.2 | 91.9 | 72.3 | 0.653 |
| Decision trees | 36.2 | 71.8 | 63.3 | 0.554 |
| Support vector machine | 71.3 | 98.8 | 91.7 | 0.920 |


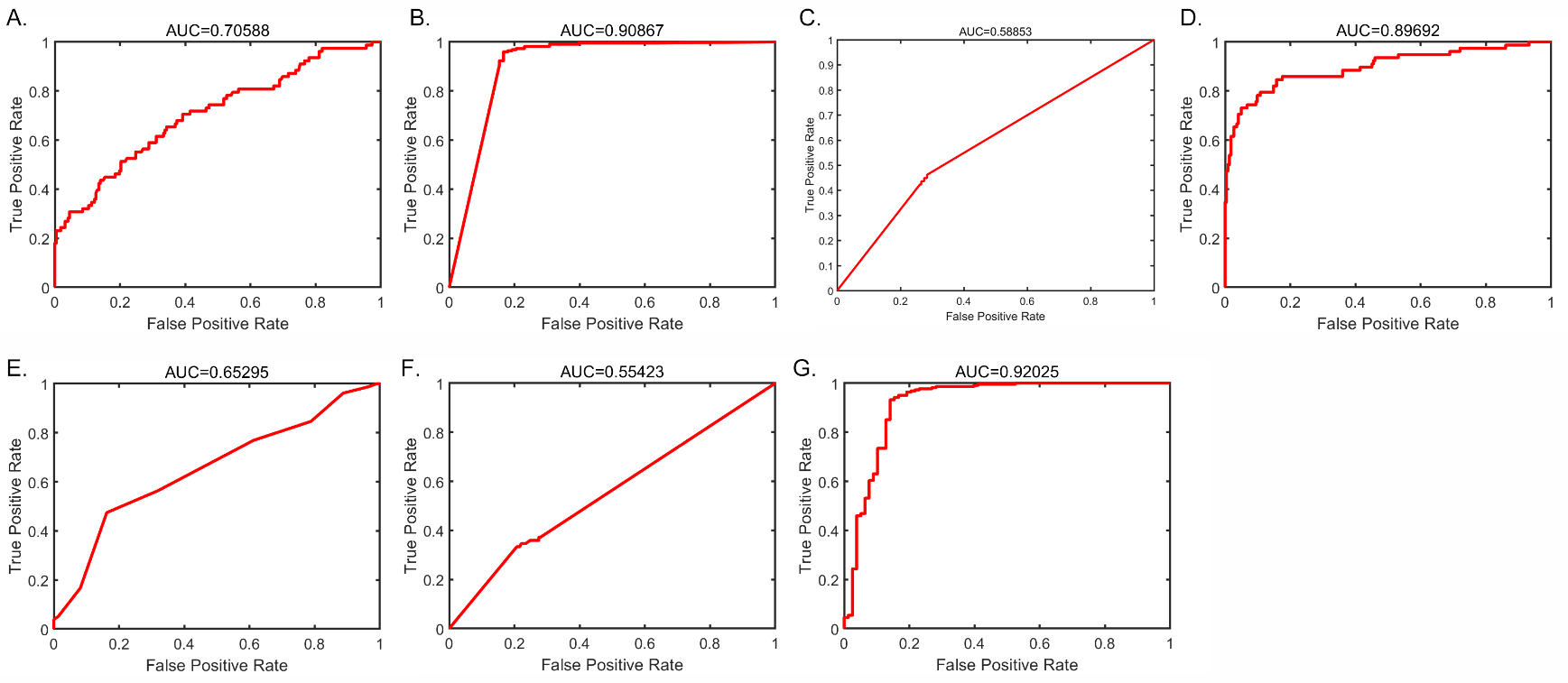


**Supplementary Figure 1** Receiver operating characteristic curve (ROC) for the support vector machine results of multiple classification model in training set (A. Random Forest, B. Logistic regression, C. Naïve Bayes, D. Linear discriminant analysis, E. K-nearest neighbors, F. Decision trees, G. Support vector machine).
